# The Impact of Neoadjuvant Chemoradiation Therapy on Non-Tumorous Barrett’s Dysplasia of the Esophagus: A Multicenter Cohort Study

**DOI:** 10.3390/jcm15010285

**Published:** 2025-12-30

**Authors:** Vismaya S. Bachu, Jay M. Lee, Hanlin L. Wang, Phillip Kozan, Melanie Ramirez, Jose Garcia-Corella, Kevin A. Ghassemi, Venkataraman Muthusamy, Danny Issa

**Affiliations:** 1 Department of Medicine, David Geffen School of Medicine, University of California Los Angeles, Los Angeles, CA 90095, USA; vbachu@mednet.ucla.edu (V.S.B.); meramirez@mednet.ucla.edu (M.R.); 2 Thoracic Oncology Program, Division of Thoracic Surgery, University of California Los Angeles, Los Angeles, CA 90095, USA; jaymoonlee@mednet.ucla.edu; 3 Department of Pathology & Laboratory Medicine, David Geffen School of Medicine, University of California Los Angeles, Los Angeles, CA 90095, USA; hanlinwang@mednet.ucla.edu; 4 Vatche and Tamar Manoukian Division of Digestive Diseases, Department of Medicine, David Geffen School of Medicine, University of California Los Angeles, Los Angeles, CA 90095, USA; pkozan@mednet.ucla.edu (P.K.); kghassemi@mednet.ucla.edu (K.A.G.); raman@mednet.ucla.edu (V.M.); 5 Internal Medicine, University of California Los Angeles (UCLA)–Kern Medical, Bakersfield, CA 93305, USA; jose.garciacorella@kernmedical.com

**Keywords:** Barrett’s esophagus, esophageal adenocarcinoma, neoadjuvant chemoradiation therapy, dysplasia, esophagectomy, multicenter study

## Abstract

**Background/Objectives**: Barrett’s esophagus (BE) is a precursor to esophageal adenocarcinoma (EAC), and neoadjuvant chemoradiation therapy (NCRT) is commonly used in the treatment of EAC. However, the impact of NCRT on non-tumorous BE and dysplasia is poorly understood. Our study aims to evaluate the effects of NCRT on BE segment length and dysplasia in patients undergoing esophagectomy for EAC. **Methods**: This multicenter, retrospective cohort study includes EAC patients who underwent esophagectomy with or without NCRT between 2014 and 2020. Patients with histologically confirmed BE and dysplasia (low- or high-grade) were analyzed. Preoperative and postoperative pathology were compared to assess BE regression, dysplastic changes, and segment length. Statistical analyses included chi-square and *t*-tests, with *p* < 0.05 considered significant. **Results**: Of 101 patients who were diagnosed with EAC, 28 patients were found to have BE, with 18 receiving NCRT in addition to surgery and 10 undergoing surgery alone. The NCRT group showed significantly higher BE regression than the control group (77.8% versus 10%, *p* < 0.001). Regression of dysplasia occurred in 66.7% of the NCRT group versus 20% of the control group (*p* = 0.079) and residual dysplasia was lower in the NCRT group (33.3%) compared to the control group (80%) (*p* = 0.018). **Conclusions**: NCRT significantly reduces BE and dysplasia, suggesting it may improve surgical outcomes by minimizing residual disease. These findings support the potential of NCRT to enhance surgical precision in EAC treatment, though further research is needed to explore underlying mechanisms and refine treatment strategies.

## 1. Introduction

### 1.1. Epidemiology and Clinical Significance

Barrett’s esophagus (BE), defined by the metaplastic transformation of distal esophageal squamous epithelium into specialized columnar epithelium, is the established precursor to esophageal adenocarcinoma (EAC). Both BE and EAC have risen sharply in Western countries over recent decades [1,2]. EAC incidence surged in the 1990s (APC 8.2%) and has continued to rise more slowly over the following years (~0.5% per year from 1999–2019) [3]. Despite improved detection methods and advancements in endoscopy, outcomes remain poor, with a 5-year survival of approximately 20% [4]. Much of this mortality burden stems from the asymptomatic nature of EAC until it reaches an advanced stage, which contributes to roughly 40% of diagnoses occurring only after the disease has already advanced.

Patients with BE have an estimated 3–5% lifetime risk of progression to EAC [5]. The American College of Gastroenterology (ACG) updated BE guidelines in 2023, recommending a single endoscopy for patients with chronic gastroesophageal reflux disease (GERD) symptoms who have ≥3 risk factors (male sex, white race, tobacco use, obesity, family history) [6]. Even with clear risk stratification, practical implementation of screening guidelines and protocols is challenging, which can hinder early detection efforts and may negatively impact patient outcomes [7]. Regarding treatment, although proton pump inhibitors effectively suppress gastric acid and alleviate reflux-related symptoms, non-acid components of reflux, including pepsin and trypsin, may continue to exert injurious effects on the esophageal mucosa and contribute to the development and persistence of BE.

### 1.2. Diagnosis and Risk Stratification

BE is diagnosed via endoscopy and biopsy according to the Seattle protocol, which recommends eight systematic samples [6]. Segments ≥3 cm are classified as long-segment BE, and <3 cm as short-segment BE. To standardize documentation, the Prague classification is recommended when reporting Barrett’s esophagus on endoscopy. This system records both the circumferential length of metaplasia and the maximal proximal extent of disease [8]. Dysplasia ranges from nondysplastic through low-grade (LGD) and high-grade (HGD) dysplasia, up to adenocarcinoma. The management of BE depends heavily on the severity of dysplasia. HGD is treated with endoscopic eradication therapy (EET), including endoscopic resection and radiofrequency ablation, while LGD is often treated with EET as well, however it warrants shared decision-making regarding watchful surveillance and follow up post-EET [9]. In selected cases, endoscopic ultrasound may further aid in evaluating deeper layers of the esophageal wall and regional lymph nodes, particularly when there is concern for submucosal invasion or malignant transformation.

### 1.3. Esophageal Adenocarcinoma Management

Once malignant transformation occurs, treatment shifts from endoscopic management of dysplasia to oncologic staging and resection. Across multiple studies, the prevalence of esophageal adenocarcinoma among patients with Barrett’s esophagus has been estimated at around 3% (95% CI, 2–5%) [10]. The management of EAC is determined by depth of invasion and associated risk of lymph node involvement, as lymph node metastasis is proven to be associated with poorer prognosis [11]. With a low risk of lymph node metastasis, intramucosal (T1a) adenocarcinoma is managed similarly to the management of HGD, with EET followed by ablation [12]. Submucosal (T1b) EAC, however, exhibits a greater risk of lymph node metastasis (up to 30% according to studies) and is preferentially managed with esophagectomy [13]. Management of locally invasive EAC (≥T2) has largely focused on surgery for resectable disease, namely radical esophagectomy, combined with neoadjuvant or perioperative chemotherapy is standard [12].

BE segment length and dysplasia degree guide surgical extent. Each additional centimeter of BE increases the odds of EAC by approximately 25% (95% CI, 1.16–1.36) [14]. Longer segments and HGD often require more extensive resection due to microscopic lateral and submucosal spread, which complicates margin assessment during surgery [15]. Achieving negative (R0) resection margins is critical, with generous margins associated with substantially higher 5-year survival compared with positive (R1) margins [16].

### 1.4. Neoadjuvant Chemoradiation Therapy (NCRT)

Given the high rate of recurrence following esophagectomy, neoadjuvant chemoradiation therapy (NCRT) has become a cornerstone in treating locally advanced esophageal cancer [17]. NCRT increases the likelihood of R0 resection, particularly in locally advanced disease. The landmark CROSS trial showed NCRT (carboplatin and paclitaxel plus radiotherapy) markedly improved surgical and survival outcomes compared to esophagectomy alone, significantly increasing resection rates to 92% compared with 69% and reduced the risk of death by roughly one-third (HR 0.66) [18]. Combination chemoradiotherapy using carboplatin–paclitaxel or cisplatin–fluorouracil has been adopted as a standard therapeutic approach in the United States [19], though recent European studies suggest that Fluorouracil, Leucovorin, Oxaliplatin, and Docetaxel (FLOT) therapy may be preferable for gastroesophageal cancers [20]. However, while these regimens are known to shrink tumor burden, their effect on noncancerous BE epithelium and dysplastic lesions remains unclear and not studied. Pathologists and surgeons may incidentally observe regression, but data quantifying these changes are sparse.

### 1.5. Rationale and Aim

Determining whether NCRT induces regression of BE or dysplasia has important implications for surgical planning, intraoperative margin assessment, residual disease, and recurrence risk. While neoadjuvant chemoradiation therapy is well established for locally advanced esophageal adenocarcinoma, its impact on precursor lesions such as Barrett’s esophagus and dysplasia has not been systematically evaluated. Our study aims to address this gap in literature by evaluating changes in BE segment length by examining the effects of NCRT on BE segment length and dysplasia in patients undergoing esophagectomy for esophageal adenocarcinoma. Given the relative rarity of patients with well-documented pre- and post-treatment histology of non-tumorous Barrett’s mucosa, existing data are limited to small retrospective cohorts. Accordingly, this study was designed as a multicenter retrospective analysis intended to generate hypothesis-driven insights into the histologic effects of neoadjuvant therapy on Barrett’s esophagus in patients undergoing esophagectomy for esophageal adenocarcinoma.

## 2. Materials and Methods

### 2.1. Study Population

**Figure 1:**
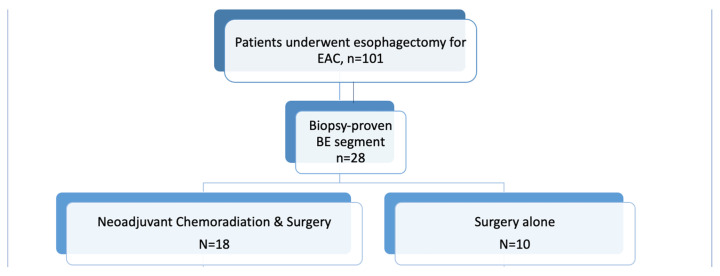
Flow chart representing patient selection and study groups. *BE*, *Barrett’s esophagus*; *EAC*, *Esophageal Adenocarcinoma*.

This multicenter, retrospective cohort study was conducted across two tertiary care referral medical centers in the United States, both of which are high-volume institutions with established multidisciplinary esophageal cancer programs. These centers were selected for their comprehensive databases and consistent histopathologic reporting practices, allowing for standardized data collection across sites. We identified adult patients diagnosed with EAC who underwent esophagectomy with or without NCRT from January 2014 to October 2020. All esophagectomies were performed at high-volume tertiary referral centers, predominantly using minimally invasive or hybrid minimally invasive approaches in accordance with contemporary surgical standards [21]. Inclusion criteria were adults with EAC who underwent esophagectomy, biopsy-proven BE with histologic evaluations confirming intestinal metaplasia and the presence of either LGD or HGD within two years prior to the surgical intervention, and pathological evaluation of surgical specimens post-operatively [Figure 1]. Patients were excluded if they lacked complete preoperative or postoperative pathology results, including reports without documentation of non-tumorous esophageal mucosa.

### 2.2. Data Collection

Preoperative data were obtained from biopsy samples collected prior to NCRT (for the NCRT group) or immediately before surgery (for the control group). All tissue specimens were reviewed by board-certified gastrointestinal pathologists using standardized diagnostic criteria to identify LGD and HGD, ensuring consistent classification across institutions. Each sample was evaluated for the presence, extent, and severity of dysplasia, as well as for any inflammatory or regenerative changes that could influence interpretation. Clinical and pathologic staging of esophageal adenocarcinoma was determined according to the TNM staging model and was extracted from multidisciplinary tumor board documentation and operative pathology reports.

Postoperative assessments involved detailed analysis of esophagectomy specimens to evaluate changes in BE segments and adjacent non-neoplastic mucosa. Examinations focused on identifying histologic regression, such as resolution or downgrading of dysplasia, or progression, indicated by higher-grade lesions or expansion of metaplastic epithelium. When available, preoperative and postoperative findings were compared directly to determine the degree of histologic change associated with neoadjuvant therapy or surgical resection.

### 2.3. Outcomes and Statistical Analysis

The primary outcome was BE regression, defined as a histologic change from HGD to LGD or complete resolution of dysplasia or metaplasia in both the NCRT and control groups. This outcome was selected to assess potential histologic improvement associated with neoadjuvant therapy compared with surgery alone. Secondary outcomes included changes in the measured length of the BE segment and any evidence of disease progression, defined as a shift from metaplasia to dysplasia or from LGD to HGD on postoperative evaluation. Exploratory analyses also examined potential associations between regression or progression and patient demographics or treatment-related factors.

Descriptive statistics were used to summarize patient demographics, clinical characteristics, and histologic findings. Comparisons between the NCRT and control groups were performed using chi-square tests for categorical variables and independent-sample *t*-tests for continuous variables after verifying normality assumptions. Rates of BE and dysplasia regression were compared using Fisher’s exact or chi-square tests, as appropriate. A *p*-value of <0.05 was considered statistically significant for all analyses. Statistical analyses were conducted using Microsoft Excel.

## 3. Results

### 3.1. Study Cohort

A total of 101 patients with EAC who underwent surgical resection between 2014 and 2020 met the study’s inclusion criteria. Among these, 28 patients were found to have concurrent BE based on preoperative or pathologic evaluation. The mean age at diagnosis was 68.2 years (±7.7), and the majority of patients were male (78.6%), consistent with the known epidemiologic distribution of EAC. Of the 28 patients with BE, 20 had long-segment BE, while 17 demonstrated dysplasia within the non-tumorous segments of BE. Among these, 14 had HGD and 3 had LGD on histologic assessment. The study population was divided into two groups: 18 patients who received NCRT followed by esophagectomy (treatment group) and 10 patients who underwent esophagectomy alone without neoadjuvant therapy (control group) [Figure 1].

### 3.2. Demographic and Clinical Characteristics

The treatment group and the control group were generally similar with respect to age (68.4 vs. 72.7 years, *p* > 0.05), gender distribution (male 83.3% vs. 70%, *p* = 0.41), and overall ethnic backgrounds (*p* = 0.57), indicating comparable baseline characteristics between the two cohorts. Notably, there was a higher proportion of white patients in the control group (90%) compared to the NCRT group (50%, *p* = 0.03), reflecting a statistically significant difference in racial composition. Health assessment scores, including the American Society of Anesthesiologists (ASA) physical status classification and Charlson Comorbidity Index (CCI), were similar between the two groups, suggesting similar overall preoperative health status. Other demographic and baseline clinical characteristics, including comorbidities, body mass index, and smoking history, are summarized in Table 1, providing a comprehensive overview of the study population.

### 3.3. Treatment Characteristics

Chemotherapy regimens in the NCRT group consisted primarily of Carboplatin plus Taxol, administered to 83.3% of patients, while smaller proportions received either Cisplatin plus 5-Fluorouracil (5-FU) (11.1%) or the combination regimen of Fluorouracil, Leucovorin, Oxaliplatin, and Docetaxel (5.6%). Radiation therapy was delivered concurrently with chemotherapy, with patients in the NCRT cohort undergoing an average of 5.3 cycles (±1.99) prior to surgical resection. Baseline assessment of BE demonstrated that the NCRT group had a mean maximum BE length of 4.7 cm (±3.2), which was shorter than the 7.5 cm (±3.5) observed in the control group (*p* = 0.03). The circumferential extent of BE was 4.2 cm ± 4.5 in the NCRT group versus 5.7 cm ± 4.4 in the control group (*p* = 0.13) [Table 1].

### 3.4. Outcomes

In the NCRT group, 14 of 18 patients (77.8%) achieved histologic regression of BE disease, while this outcome was only seen in 1 of 10 patients (10%) in the control group (*p* < 0.001). Conversely, no patients in the NCRT group experienced BE progression, whereas 7 out of 10 patients in the control group exhibited significant progression of Barrett’s disease (*p* < 0.001) [Table 2].

Regarding the level of dysplasia before esophagectomy, both patient populations had similar rates of LGD and HGD. Postoperatively, however, residual dysplasia was significantly less common in the NCRT group, observed in 6 of 18 patients (33.3%) versus 8 of 10 patients (80%) in the control group (*p* = 0.018). This difference was primarily driven by residual HGD, present in 3 of 18 patients (16.7%) in the NCRT group compared to 6 of 10 patients (60%) in the control group (*p* = 0.019), whereas residual LGD was similar between the groups (3/18, 16.7% vs. 2/10, 20%).

**Figure 2:**
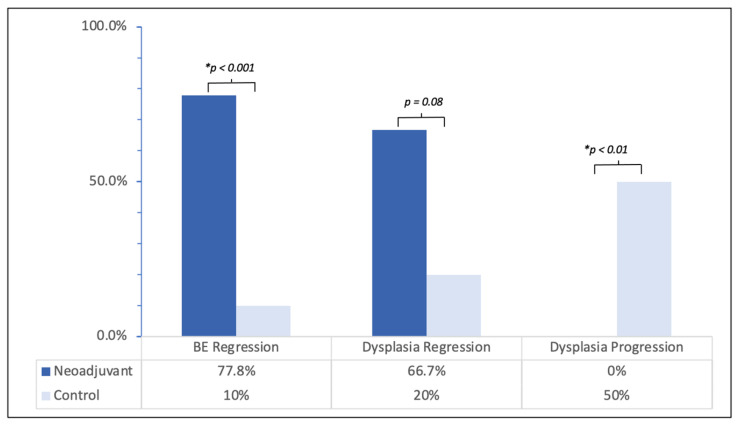
Regression of Barrett’s esophagus and regression of dysplasia in the neoadjuvant vs. control group. BE, Barrett’s esophagus. * *p* < 0.05.

Regression of dysplasia was more frequent following NCRT, occurring in 8 of 12 patients (66.7%) versus 1 of 5 patients (20%) in the control group (*p* = 0.079). Specifically, regression of HGD was noted in 7 of 10 patients (70%) in the NCRT group compared with 1 of 4 patients (25%) in the control group (*p* = 0.12). Overall, postoperative residual dysplasia remained in one-third of NCRT patients, contrasting with 80% of controls, underscoring the impact of NCRT on dysplasia reduction [Figure 2].

## 4. Discussion

This study demonstrates that neoadjuvant chemoradiation therapy induces high rates of regression in non-tumorous BE segments in patients undergoing surgical resection for EAC. Specifically, 77.8% of patients receiving NCRT showed BE regression on postoperative pathology compared to only 10% in the control group, underscoring the potential of NCRT to downstage BE and reduce residual dysplasia. Our findings are particularly notable given that both groups had similar baseline rates of LGD and HGD prior to intervention, suggesting that the observed differences may be attributable to the effect of NCRT rather than the burden of initial disease.

### 4.1. Comparison with Previous Literature

Though the role of NCRT in improving outcomes of locally advanced esophageal and gastroesophageal junction cancers is clear, current literature on its impact on BE and different grades of dysplasia is limited [22]. One of the earliest studies on the subject by Girling et al. in 2002 examined 802 patients with resectable EAC [23]. Compared to 402 patients who underwent surgery alone, the 400 patients who were also administered neoadjuvant chemotherapy (cisplatin + 5-FU) had significantly increased surgical margins without tumor (60% vs. 54%), increased median survival time (16.8 months vs. 13.3 months), and increased 2-year survival rate (43% vs. 34%). An update to the trial published in 2009 by Allum et al. also noted a significant increase (*p* = 0.03) in 5-year overall survival (OS) of patients who were administered neoadjuvant chemotherapy (23%) versus those who underwent surgery alone (17%) [24]. Further, preoperative NCRT was demonstrated to decrease locoregional recurrence in the CROSS trial by Oppedijk et al. (2014) cementing the role of NCRT in addition to surgical resection in the treatment of EAC [18]. Our findings build upon this prior work, providing novel insight into the impact of NCRT on non-tumorous BE segments.

In contrast, the study done by Barthel et al. suggests there is persistence of BE with unchanged length in patients who receive NCRT; however, a few key differences are worth noting [25]. First, there was no stratification by level of dysplasia, with the study rather measuring presence or absence of BE after esophagectomy, precluding the assessment of dysplasia regression. Secondly, their study included patients who underwent NCRT and definitive chemoradiation therapy, but no control group; this is particularly important as we highlight not only lack of dysplasia regression, but also an increased rate of disease progression in the population treated with surgery alone. Lastly, their NCRT regimen consisted of 2 cycles of cisplatin and 5-Fluorouracil for all patients, whereas our cohort included various regimens, with predominance of carboplatin and paclitaxel.

### 4.2. Clinical Implications

Our results carry particular clinical relevance for patients with early-stage EAC and long-segment BE or HGD. Patients undergoing surgery alone are at substantially higher risk for residual BE and dysplasia, highlighting the need for postoperative endoscopic surveillance. In such patients, additional interventions including endoscopic ablation or further resection may be warranted to mitigate future malignant transformation. Conversely, patients treated with NCRT appear to have a reduced risk of residual BE and progression, suggesting that preoperative NCRT may favorably modulate the histologic landscape of BE. Importantly, patients receiving neoadjuvant chemoradiation generally had more advanced clinical stage disease at presentation, reflecting guideline-based treatment allocation rather than selection bias. Despite this, the NCRT cohort demonstrated significantly greater regression of Barrett’s esophagus and dysplasia, suggesting that the observed histologic effects are unlikely to be explained solely by baseline disease severity. These findings reinforce the importance of preoperative endoscopic evaluation and targeted biopsies after NCRT but prior to surgery to inform surgical planning, potentially allowing for more precise resections, organ preservation, and reduced surgical morbidity.

### 4.3. Biological Considerations and Mechanistic Insights

The observed regression of BE and dysplasia following NCRT raises important questions regarding the biological mechanisms underlying treatment response in non-tumorous tissue. While tumor cells are known to be highly radiosensitive due to their rapid proliferation and genomic instability, non-neoplastic Barrett’s epithelium may also exhibit sensitivity to DNA damage induced by chemoradiation [26]. Experimental studies suggest that ionizing radiation and chemotherapeutic agents can induce apoptosis and cell cycle arrest in metaplastic epithelium, potentially contributing to regression of dysplasia [27]. Additionally, chemoradiation may modulate the local immune microenvironment, enhancing antitumor and anti-dysplastic responses. Radiation-induced cytokine release and immune cell recruitment could facilitate clearance of dysplastic cells, an effect that warrants further molecular investigation [28]. Understanding these mechanisms could inform the development of targeted neoadjuvant therapies that maximize regression of precursor lesions while minimizing toxicity to normal esophageal mucosa.

### 4.4. Challenges and the Role of Preoperative Planning

However, even with these potential benefits of NCRT and careful preoperative planning, challenges remain in achieving optimal long-term outcomes due to persistent disease recurrence and the limitations of surgical intervention alone. Despite improved locoregional control of disease [18], higher rates of negative surgical margins [29], and increased pathologic complete response in patients treated with a multimodal regimen [30,31], around 20% of patients still experience disease recurrence, leading to more radical surgical resection techniques to ensure comprehensive tumor removal. Given the limited efficacy of salvage therapy in these patients [32,33] and underexplored role of NCRT in the treatment of BE and dysplasia to improve surgical margins, our above findings especially underscore the importance of incorporating preoperative endoscopic biopsies following NCRT but before surgical resection. These biopsies could be essential for assessing BE regression and adjusting surgical strategies to achieve precise resections, which could avoid repeat procedures and increase organ preservation. This approach may lead to reduced surgical morbidity and improved patient outcomes.

### 4.5. Limitations

This study has some limitations that merit discussion. The small sample size and retrospective design may limit generalizability and introduce bias. These constraints reflect the stringent inclusion criteria required for well-characterized pre- and post-treatment histologic assessment of non-tumorous Barrett’s esophagus across multiple institutions. While most patients received similar chemoradiation regimens, variability in treatment protocols could have influenced the response. Additionally, potential confounding factors, such as tumor biology and immunologic profiles, were not routinely assessed during the study and may have affected outcomes. The impact of perioperative chemotherapy alone on esophageal dysplasia is unclear and warrants further exploration, particularly given its growing role in resectable esophageal cancer (ESOPEC trial) [34]. Moreover, the molecular mechanisms underlying BE’s response to NCRT remain poorly understood, which limits our ability to fully explain our findings. Lastly, the emerging role of adjuvant immune checkpoint inhibition in dysplasia regression (CheckMate 577) presents an opportunity to explore its potential effects on dysplasia regression, either alone or in combination with NCRT [35]. Our findings should be interpreted as hypothesis-generating and warrant validation in larger, prospective cohorts.

### 4.6. Summary and Future Directions

Taken together, our findings suggest that NCRT not only improves outcomes for primary locally advanced EAC, including overall survival, disease-free survival, and resection rates, but may also promote meaningful regression of non-tumorous BE segments in high-risk patients with long-segment BE or HGD. These results underscore the importance of incorporating histologic assessment of BE and dysplasia into preoperative and perioperative planning, in line with specialty society recommendations for expert pathology review, systematic biopsies, and risk stratification. Future research should prioritize evaluation of long-term outcomes, including recurrence, progression, and quality of life. In addition, studies incorporating advanced immunohistochemical and molecular profiling may provide mechanistic insight into the observed regression of BE and dysplasia, although such analyses were not routinely available in this retrospective cohort. Collectively, these efforts aim to refine patient selection, enable risk-adapted therapy, and advance personalized, organ-preserving strategies for the management of EAC and its precursor lesions.

## 5. Conclusions

In summary, NCRT significantly impacts BE and leads to dysplasia regression while reducing disease progression in the non-tumorous Barrett’s segments, suggesting that NCRT could improve outcomes in EAC arising from BE by enabling more precise resections and minimizing residual disease. Given these findings, incorporating post-NCRT biopsies into preoperative planning may enhance surgical precision, increase rates of organ preservation, and potentially reduce morbidity caused by extensive or recurring procedures. These results also highlight the potential for NCRT to modulate the histologic environment of BE, which could inform risk stratification and personalized treatment approaches. Future research is essential to determine the mechanisms behind BE’s response to chemoradiation therapy, evaluate long-term outcomes, and refine treatment strategies accordingly.

## Figures and Tables

**Table 1:** Demographic and baseline characteristics of our neoadjuvant group and control groups. *5-Fluorouracil (5-FU)*; *Fluorouracil*, *Leucovorin*, *Oxaliplatin,* and *Docetaxel (FLOT)*.

	NCRT + Surgery (N = 18)	Surgery Alone (N = 10)	*p* Value
Age (years)	68.4 ± 7.1	72.7 ± 7.8	0.064
Gender, n (%)			0.41
Male	15 (83.3)	7 (70)	
Female	3 (16.7)	3 (30)	
Race, n (%)			0.03
White	9 (50)	9 (90)	
Other	9 (50)	0 (0)	
Declined	0 (0)	1 (10)	
Ethnicity, n (%)			0.57
Not Hispanic	10 (55.6)	7 (70)	
Hispanic	7 (38.9)	2 (20)	
Other	1 (5.50)	1 (10)	
Health Class, Mean ± SD			
ASA	2.5 ± 0.79	2.4 ± 0.52	0.39
CCI	4.82 ± 1.62	5.1 ± 0.74	0.28
BE Length, Mean ± SD			
Circumferential Length (cm)	4.20 ± 4.12	5.70 ± 4.43	0.13
Maximum Length (cm)	4.70 ± 3.20	7.51 ± 3.52	0.03
Chemotherapy		N/A	N/A
Carboplatin + Taxol, n (%)	15 (83.3)		
Cisplatin + 5-FU, n (%)	2 (11.1)		
FLOT, n (%)	1 (5.6)		
Radiation		N/A	N/A
Cycles, Mean ± SD	5.30 ± 1.99		

**Table 2:** Barrett’s esophagus and dysplasia outcome summary.

	NCRT + Surgery (N = 18)	Surgery Alone (N = 10)	*p* Value
BE Changes, n (%)			
Total BE Regressed	14 (77.8)	1 (10)	<0.001
BE Progression	0 (0)	7 (70)	<0.001
Pre-EA Dysplasia, n (%)			
Low Grade	2 (16.7)	1 (20)	0.92
High Grade	10 (83.3)	4 (80)	0.62
Post-EA Dysplasia, n (%)			
Residual Dysplasia	6 (33.3)	8 (80)	0.018
Residual LGD	3 (16.7)	2 (20)	0.3
Residual HGD	3 (16.7)	6 (60)	0.019
	**NCRT + Surgery** **(n = 12)**	**Surgery Alone with** **Dysplasia (n = 5)**	***p* Value**
Dysplasia Changes, n (%)			
Dysplasia Regression	8 (66.7)	1 (20)	0.079
HGD Regression	7 (70)	1 (25)	0.12
Dysplasia Progression	0 (0)	5 (62.5)	<0.01

## Data Availability

The raw data supporting the conclusions of this article can be made available by the authors on request.

## Abbreviations

The following abbreviations are used in this manuscript:

BE	Barrett’s esophagus
EAC	Esophageal adenocarcinoma
NCRT	Neoadjuvant chemoradiation therapy
HGD	High-grade dysplasia
LGD	Low-grade dysplasia
ASA	American Society of Anesthesiologists
CCI	Charlson Comorbidity Index
5-FU	5-Fluorouracil
CROSS	Chemoradiotherapy for Oesophageal cancer followed by Surgery Study
ESOPEC	Esophageal cancer trial

## References

[1] Pohl H., Welch H.G. (2005). The role of overdiagnosis and reclassification in the marked increase of esophageal adenocarcinoma incidence. J. Natl. Cancer Inst..

[2] Thrift A.P. (2021). Global burden and epidemiology of Barrett oesophagus and oesophageal cancer. Nat. Rev. Gastroenterol. Hepatol..

[3] Xie Z., Lin J., Li Z., Sun H., Huang K., Lin D., Xiao Y., Li C., Zeng D. (2025). Esophageal cancer trends in the US from 1992 to 2019 with projections to 2044 using SEER data. Sci. Rep..

[4] Codipilly D.C., Sawas T., Dhaliwal L., Johnson M.L., Lansing R., Wang K.K., Leggett C.L., Katzka D.A., Iyer P.G. (2021). Epidemiology and Outcomes of Young Onset Esophageal Adenocarcinoma: An Analysis from a Population-Based Database. Cancer Epidemiol. Biomark. Prev..

[5] Eusebi L.H., Cirota G.G., Zagari R.M., Ford A.C. (2021). Global prevalence of Barrett’s oesophagus and oesophageal cancer in individuals with gastro-oesophageal reflux: A systematic review and meta-analysis. Gut.

[6] Shaheen N.J., Falk G.W., Iyer P.G., Souza R.F., Yadlapati R.H., Sauer B.G., Wani S. (2022). Diagnosis and Management of Barrett’s Esophagus: An Updated ACG Guideline. Am. J. Gastroenterol..

[7] Eluri S., Reddy S., Ketchem C.C., Tappata M., Nettles H.G., Watts A.E., Cotton C.C., Dellon E.S., Shaheen N.J. (2022). Low Prevalence of Endoscopic Screening for Barrett’s Esophagus in a Screening-Eligible Primary Care Population. Am. J. Gastroenterol..

[8] Sharma P., Dent J., Armstrong D., Bergman J.J., Gossner L., Hoshihara Y., Jankowski J.A., Junghard O., Lundell L., Tytgat G.N. (2006). The development and validation of an endoscopic grading system for Barrett’s esophagus: The Prague C & M criteria. Gastroenterology.

[9] Rubenstein J.H., Shaheen N.J. (2015). Epidemiology, Diagnosis, and Management of Esophageal Adenocarcinoma. Gastroenterology.

[10] Parasa S., Desai M., Vittal A., Chandrasekar V.T., Pervez A., Kennedy K.F., Gupta N., Shaheen N.J., Sharma P. (2019). Estimating neoplasia detection rate (NDR) in patients with Barrett’s oesophagus based on index endoscopy: A systematic review and meta-analysis. Gut.

[11] Cabau M., Luc G., Terrebonne E., Belleanne G., Vendrely V., Cunha A.S., Collet D. (2013). Lymph node invasion might have more prognostic impact than R status in advanced esophageal adenocarcinoma. Am. J. Surg..

[12] Schlottmann F., Patti M.G., Shaheen N.J. (2017). Endoscopic Treatment of High-Grade Dysplasia and Early Esophageal Cancer. World J. Surg..

[13] Pennathur A., Farkas A., Krasinskas A.M., Ferson P.F., Gooding W.E., Gibson M.K., Schuchert M.J., Landreneau R.J., Luketich J.D. (2009). Esophagectomy for T1 esophageal cancer: Outcomes in 100 patients and implications for endoscopic therapy. Ann. Thorac. Surg..

[14] Krishnamoorthi R., Singh S., Ragunathan K., Visrodia K., Wang K.K., Katzka D.A., Iyer P.G. (2018). Factors Associated With Progression of Barrett’s Esophagus: A Systematic Review and Meta-analysis. Clin. Gastroenterol. Hepatol..

[15] Pech O., May A., Manner H., Behrens A., Pohl J., Weferling M., Hartmann U., Manner N., Huijsmans J., Gossner L. (2014). Long-term efficacy and safety of endoscopic resection for patients with mucosal adenocarcinoma of the esophagus. Gastroenterology.

[16] Pang T., Nie M., Yin K. (2023). The correlation between the margin of resection and prognosis in esophagogastric junction adenocarcinoma. World J. Surg. Oncol..

[17] Rustgi A.K., El-Serag H.B. (2014). Esophageal Carcinoma. N. Engl. J. Med..

[18] Oppedijk V., van der Gaast A., van Lanschot J.J., van Hagen P., van Os R., van Rij C.M., van der Sangen M.J., Beukema J.C., Rütten H., Spruit P.H. (2014). Patterns of recurrence after surgery alone versus preoperative chemoradiotherapy and surgery in the CROSS trials. J. Clin. Oncol..

[19] Malthaner R., Wong R.K.S., Spithoff K. (2010). Gastrointestinal Cancer Disease Site Group of Cancer Care Ontario’s Program in Evidence-based Care. Preoperative or postoperative therapy for resectable oesophageal cancer: An updated practice guideline. Clin. Oncol. (R. Coll. Radiol.).

[20] Al-Batran S.E., Homann N., Pauligk C., Goetze T.O., Meiler J., Kasper S., Kopp H.G., Mayer F., Haag G.M., Luley K. (2019). Perioperative chemotherapy with fluorouracil plus leucovorin, oxaliplatin, and docetaxel versus fluorouracil or capecitabine plus cisplatin and epirubicin for locally advanced, resectable gastric or gastro-oesophageal junction adenocarcinoma (FLOT4): A randomised, phase 2/3 trial. Lancet.

[21] Vashist Y., Goyal A., Shetty P., Girnyi S., Cwalinski T., Skokowski J., Malerba S., Prete F.P., Mocarski P., Kania M.K. (2025). Evaluating Postoperative Morbidity and Outcomes of Robotic-Assisted Esophagectomy in Esophageal Cancer Treatment—A Comprehensive Review on Behalf of TROGSS (The Robotic Global Surgical Society) and EFISDS (European Federation International Society for Digestive Surgery) Joint Working Group. Curr. Oncol..

[22] Yang H., Wang F., Hallemeier C.L., Lerut T., Fu J. (2024). Oesophageal cancer. Lancet.

[23] Medical Research Council Oesophageal Cancer Working Party (2002). Surgical resection with or without preoperative chemotherapy in oesophageal cancer: A randomised controlled trial. Lancet.

[24] Allum W.H., Stenning S.P., Bancewicz J., Clark P.I., Langley R.E. (2009). Long-term results of a randomized trial of surgery with or without preoperative chemotherapy in esophageal cancer. J. Clin. Oncol..

[25] Barthel J.S., Kucera S.T., Lin J.L., Hoffe S.E., Strosberg J.R., Ahmed I., Dilling T.J., Stevens C.W. (2010). Does Barrett’s esophagus respond to chemoradiation therapy for adenocarcinoma of the esophagus?. Gastrointest. Endosc..

[26] Raouf A., Evoy D., Carton E., Mulligan E., Griffin M., Sweeney E., Reynolds J.V. (2001). Spontaneous and inducible apoptosis in oesophageal adenocarcinoma. Br. J. Cancer.

[27] Kelly R.J., Zaidi A.H., Smith M.A., Omstead A.N., Kosovec J.E., Matsui D., Martin S.A., DiCarlo C., Werts E.D., Silverman J.F. (2018). The Dynamic and Transient Immune Microenvironment in Locally Advanced Esophageal Adenocarcinoma Post Chemoradiation. Ann. Surg..

[28] Saraggi D., Fassan M., Bornschein J., Farinati F., Realdon S., Valeri N., Rugge M. (2016). From Barrett metaplasia to esophageal adenocarcinoma: The molecular background. Histol. Histopathol..

[29] Van Hagen P., Hulshof M.C.C.M., Van Lanschot J.J.B., Steyerberg E.W., Henegouwen M.V.B., Wijnhoven B.P.L., Richel D.J., Nieuwenhuijzen G.A.P., Hospers G.A.P., Bonenkamp J.J. (2012). Preoperative chemoradiotherapy for esophageal or junctional cancer. N. Engl. J. Med..

[30] Pasini F., de Manzoni G., Zanoni A., Grandinetti A., Capirci C., Pavarana M., Tomezzoli A., Rubello D., Cordiano C. (2013). Neoadjuvant therapy with weekly docetaxel and cisplatin, 5-fluorouracil continuous infusion, and concurrent radiotherapy in patients with locally advanced esophageal cancer produced a high percentage of long-lasting pathological complete response: A phase 2 study. Cancer.

[31] Almhanna K., Hoffe S., Strosberg J., Dinwoodie W., Meredith K., Shridhar R. (2015). Concurrent chemoradiotherapy with protracted infusion of 5-fluorouracil (5-FU) and cisplatin for locally advanced resectable esophageal cancer. J. Gastrointest. Oncol..

[32] Gastroesophageal Cancer Research Surveillance and Outcomes After Curative Resection for Gastroesophageal Adenocarcinoma. https://ge-cancer.com/testimonial/surveillance-and-outcomes-after-curative-resection-for-gastroesophageal-adenocarcinoma/.

[33] Sudo K., Taketa T., Correa A.M., Campagna M.C., Wadhwa R., Blum M.A., Komaki R., Lee J.H., Bhutani M.S., Weston B. (2013). Locoregional failure rate after preoperative chemoradiation of esophageal adenocarcinoma and the outcomes of salvage strategies. J. Clin. Oncol..

[34] Hoeppner J., Lordick F., Brunner T., Glatz T., Bronsert P., Röthling N., Schmoor C., Lorenz D., Ell C., Hopt U.T. (2016). ESOPEC: Prospective randomized controlled multicenter phase III trial comparing perioperative chemotherapy (FLOT protocol) to neoadjuvant chemoradiation (CROSS protocol) in patients with adenocarcinoma of the esophagus (NCT02509286). BMC Cancer.

[35] Kelly R.J., Ajani J.A., Kuzdzal J., Zander T., Van Cutsem E., Piessen G., Mendez G., Feliciano J., Motoyama S., Lièvre A. (2021). Adjuvant Nivolumab in Resected Esophageal or Gastroesophageal Junction Cancer. N. Engl. J. Med..

